# Magnesium Alloys With Tunable Interfaces as Bone Implant Materials

**DOI:** 10.3389/fbioe.2020.00564

**Published:** 2020-06-10

**Authors:** Mostafizur Rahman, Naba K. Dutta, Namita Roy Choudhury

**Affiliations:** Chemical and Environmental Engineering, School of Engineering, RMIT University, Melbourne, VIC, Australia

**Keywords:** interfacial engineering, magnesium alloy, surface coating, corrosion, biomedical application

## Abstract

Magnesium (Mg) based biodegradable materials are a new generation orthopedic implant materials that are intended to possess same mechanical properties as that of bone. Mg alloys are considered as promising substitutes to permanent implants due to their biodegradability in the physiological environment. However, rapid corrosion rate is one of the major constraints of using Mg alloys in clinical applications in spite of their excellent biocompatibility. Approaches to overcome the limitations include the selection of adequate alloying elements, proper surface treatment, surface modification with coating to control the degradation rate. This review focuses on current advances on surface engineering of Mg based biomaterials for biomedical applications. The review begins with a description of corrosion mechanism of Mg alloy, the requirement for appropriate surface functionalization/coatings, their structure-property-performance relationship, and suitability for biomedical applications. The control of physico-chemical properties such as wettability, surface morphology, surface chemistry, and surface functional groups of the coating tailored by various approaches forms the pivotal part of the review. Chemical surface treatment offers initial protection from corrosion and inorganic coating like hydroxyapatite (HA) improves the biocompatibility of the substrate. Considering the demand of ideal implant materials, multilayer hybrid coatings on Mg alloy in combination with chemical pretreatment or inorganic HA coating, and protein-based polymer coating could be a promising technique to improve corrosion resistance and promote biocompatibility of Mg-based alloys.

## Introduction

Magnesium (Mg) based alloys are considered as a third generation biomaterials (bioactive, biodegradable, and bio-tolerant) for tissue engineering as they can act as temporary structure for tissue regeneration and eventually degrade completely in biological medium (Heublein et al., 2003; Shi et al., 2015). The first generation biomaterials are bio-inert (metallic implant, ceramic, etc.) and the second generation biomaterials are bioactive and biodegradable (bio-ceramic and polymer) (Dharmayanti et al., 2020). Mg alloys offer a significant benefit over permanent implant materials including lightweight, close to bone density (natural bone 1.80–2.00 g/cm^3^ and Mg 1.74–2.00 g/cm^3^) and high strength (tensile) to weight ratio (158 kN-m/kg) (Li and Zheng, 2013) and the mechanical properties, advantages and disadvantages of metallic implants are listed in Table 1. Although Mg alloys exhibit numerous advantages over permanent implants, however, they display uncontrolled corrosion and degradation rate in the body fluid solution. Recently, clinical trials with Mg alloy implants have been explored as bone implants (Cha et al., 2013) and cardiovascular stents (Erbel et al., 2007) such as Mg-Sr (Bornapour et al., 2015), Mg-Ca (Zeng et al., 2015), Mg-Zn (Zhang et al., 2010), Mg- rare earth (RE) (Hort et al., 2010), and Mg-based hybrid implants (Haude et al., 2013; Wong et al., 2013). Apart from FDA approval, the major limitations of using Mg alloys in biomedical applications are as follows:

- Corrosion in the physiological environment which results in evolution of hydrogen (H_2_) gas and increases extraordinarily pH value in the body fluid solution. The H_2_ embrittlement occurs by accumulation in a gas pocket around the implant and causes early implant failure,
- Uncontrolled degradation rate i.e., mismatch between implant materials and bone tissue healing in the biological environment,
- Inferior mechanical properties.

**Table 1:** Mechanical properties, advantages, and disadvantages of metallic implants (Mani et al., 2007; Moravej and Mantovani, 2011; Chen and Thouas, 2015; Pandey et al., 2020).

**Implants**	**Density (g/cm^**3**^)**	**Modulus of elasticity (GPa)**	**Advantages**	**Disadvantages**
Mg	1.74	41–45	Almost similar density and mechanical properties to that of natural bone	Rapidly corrode in the physiological environment
Mg based alloy (WE43 ASTM B107/B107M)	1.84	41–45		
Stainless steels (SS316)	8	193	High wear resistance	Higher modulus of elasticity
Co-Cr alloys (ASTM F90)	9.2	210	High strength	Causes allergic due to Co, Cr, and Ni
Ti alloys (Ti-6Al-4V ASTM grade 1)	4.4	110	High biocompatibility	Causes toxic effect due to V and Al for long term applications. Low corrosion and wear properties as compared to other permanent implants but better than Mg

**Figure 1:**
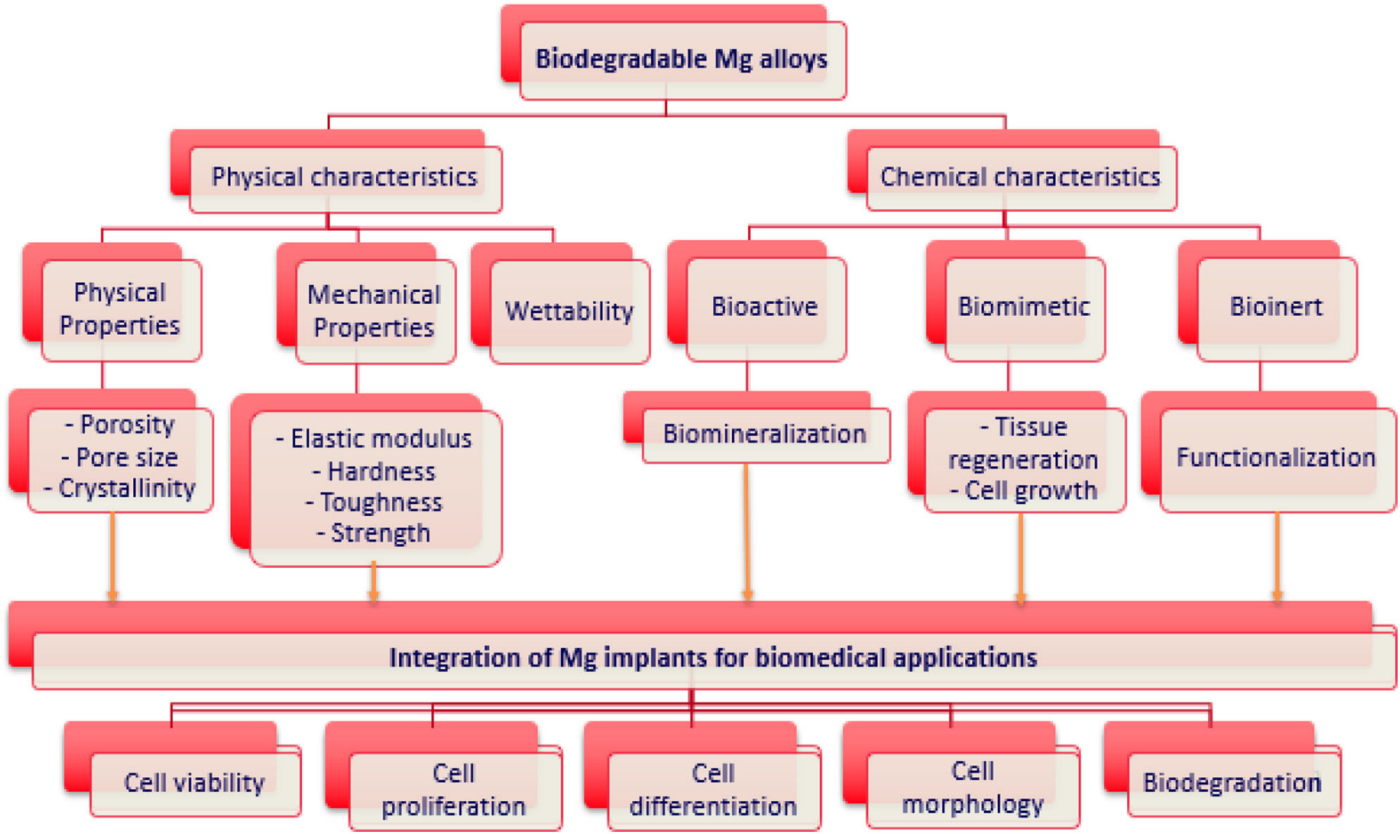
Physio-chemical properties of biodegradable Mg alloys for biomedical applications.

Approaches to overcome the above limitations include the selection of adequate alloying elements, changing microstructure, and grain size of the main alloy, the surface treatment and surface modification of the coating to control the degradation rate. Various strategies are used for corrosion inhibition including single layer, double layer, or multilayer of hydroxyapatite (HA) coating, micro-arc oxidation, sol-gel, fluoride based, and polymer coatings. These approaches in combination with inorganic- organic or hybrid coatings on biodegradable Mg-based alloys represent a fascinating strategy to enhance the corrosion properties and biomedical properties. This review will focus on the strategies to improve corrosion resistance and biocompatibility of Mg and its alloys using bioactive, bioinert, and biomimetic hybrid coatings to stimulate the bone tissue response for biomedical applications (Figure 1).

## Corrosion of Mg alloys

### Corrosion Mechanism

Untreated or unprotected Mg and its alloys readily corrode in the aqueous solution including physiological environment, simulated body fluid solution (SBF), and other biological solutions. Thermodynamically a change in free energy of the system occurs during corrosion:

(1) $$\begin{array}{l} \Delta \text{G}={-\text{nFE}}_{\text{o}} \end{array}$$

where, ΔG = Gibbs free energy change

n = Number of electrons exchanged in the reaction

F = Faraday's constant

E_o_ = Standard potential of the cell

Negative Gibbs free energy in this situation implies spontaneous oxidation reaction occurs in the system. Mg-based alloys immediately oxidize in contact with water because of poor thermodynamic stability and release hydrogen (H_2_) gas from water (Pourbaix, 1974). The anodic and cathodic reactions are presented as follows:

(2) $$\begin{array}{l} \text{Anodic reaction Mg}➝\text{M}{\text{g}}^{2+}+2{\text{e}}^{-} \end{array}$$

(3) $$\begin{array}{l} \text{Cathodic reaction }2{\text{H}}_{2}\text{O}➝{\text{H}}_{2}+2\text{O}{\text{H}}^{-} \end{array}$$

(4) $$\begin{array}{l} \text{Overall reaction M}{\text{g}}^{2+}+2\text{O}{\text{H}}^{-}➝\text{Mg}{\left(\text{OH}\right)}_{2} \end{array}$$

**Figure 2:**
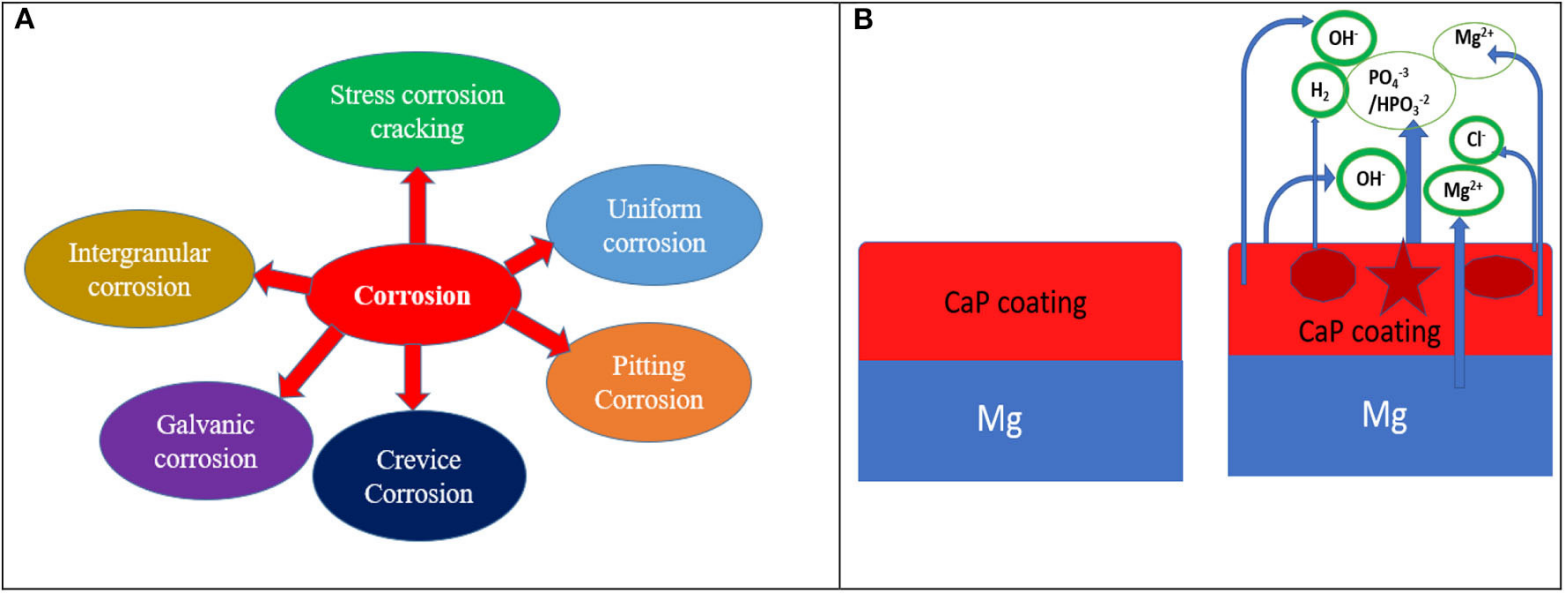
**(A)** Several forms of corrosion and **(B)** corrosion mechanism of CaP coated Mg substrate in physiological environment.

There are two main reasons for low corrosion resistance of Mg-based implant materials as (i) the deposited oxide or hydroxide films on the surface of Mg alloys are not enough protective and (ii) bimetallic or galvanic corrosion can occur because of the impurities and second phases including Mg_12_Nd (Guo et al., 2005), Al_8_Mn_5_ (Eliezer et al., 2003), and so on. There are several types of corrosion including pitting corrosion, galvanic corrosion, crevice corrosion, intergranular corrosion found in Mg-based implant materials in the biological environment and their corrosion mechanisms are presented in Figure 2A. Such corrosion can be controlled by taking some strategies such as appropriate design of alloy including alloying elements during alloy fabrication, changing surface microstructure like porosity or size of the grain, and surface modifications such as chemical treatments/coating or combination of both as shown in Figure 2B. The effect of an alloying element such as strontium (0.25% Sr) has shown to influence the microstructure, corrosion performance of a Mg-Zn-Mn alloy (Jiang et al., 2019) along with better cell adhesion and proliferation. Pre-treatment is also an effective way of changing its microstructure and composition or both to improved protection capacity when combined with a distinct coating layer of calcium phosphate (CaP), polymer or combination of both on the pre-treated surface thus, enhanced biological properties of the implant materials. When CaP coated Mg substrate is immersed in SBF solution, it starts reacting and discharges Ca^2+^ and ${\text{PO}}_{4}^{3-}$/${\text{HPO}}_{3}^{2-}$ and Cl^−^ from SBF solution. In the physiological environment, bare Mg substrate reacts with water and releases Mg^2+^ and OH^−^ and finally produces Mg(OH)_2_ as shown in reaction schemes (2) to (4) and Figure 2B. From the basic principle of corrosion, it is evident that corrosion of Mg alloy can only be slowed down to suit the need. To inhibit the reaction, either the cathodic reaction/or anodic reaction could be arrested or a large resistance in the electrochemical circuit could be included so that it can impede the movement of ions.

### Corrosion and Cell Behavior Control

**Figure 3:**
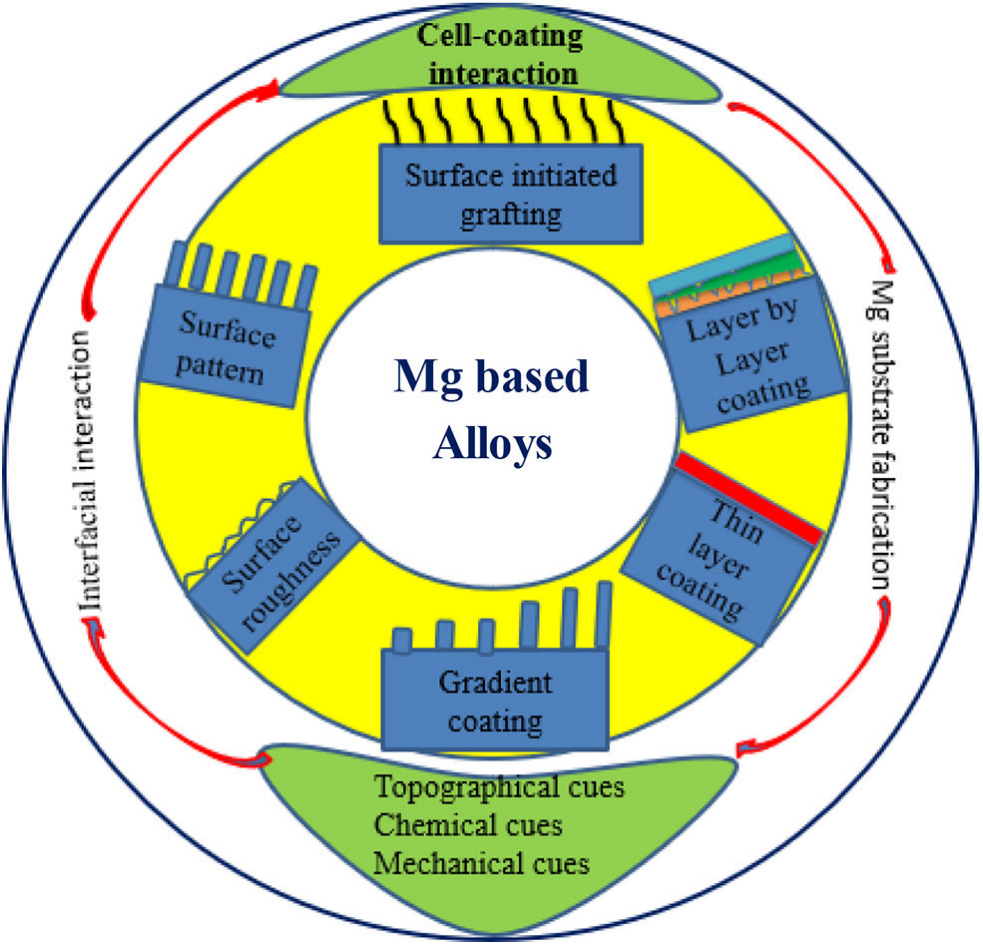
Schematic illustration of interfacial engineering of untreated Mg alloys.

Surface coating of the Mg metal is one promising approach to control corrosion. Figure 3 shows the scope of this review with potential surface/interfacial engineering approaches for Mg alloys that could tackle corrosion and cell behavior for bone TE. While the metal could be alloyed with other key metals, the intrinsic physico-chemical properties such as wettability, surface morphology, chemistry, and surface functional group of the tailored alloy can significantly control its degradation rate and bioactivity as discussed below.

#### Wettability

To establish favorable host bone tissue-biomaterials interaction, it is crucial to tailor the wettability and surface chemical properties of the Mg alloys. However, surface wettability is a key property and determined from the contact angle (CA) between the solid surface and liquid. CA reveals the wettability of the solid surface and the wettability in turn influences the cell attachment. Surface wettability is affected by both physical factors such as surface roughness/topography and chemical factors such as surface functional groups. The surface refers to hydrophilic when CA of the solid surface below 90° and surface considered to be hydrophobic when CA is more than 90° according to Young's equation. The solid surface is not perfectly flat or uniform somewhat uneven or rough and surface roughness influences the surface wettability. For example, the CA is about 39.7° on the smooth surface and CA is about 27.2° on the rough surface of Mg-Y-RE Mg alloy substrate (Jin et al., 2019). However, the hydrophobicity/hydrophilicity can be controlled by changing the topography such as surface roughness e.g, CA is about 105° on the smooth surface of ZK60 Mg alloy and CA of about 38° on the rough surface of hydroxyapatite coated ZK60 (Singh et al., 2019). The surface wettability including hydrophilicity and hydrophobicity determines the cell adhesion and cell proliferation for *in vivo* situation. Generally, the hydrophilic surfaces show better attraction for cells than hydrophobic surfaces (Lampin et al., 1997). The human skin fibroblast cells attachment on the gradient surface is seen to increase when the surface turns from the hydrophobic to hydrophilic through control of surface chemistry (Ruardy et al., 1995).

#### Surface Morphology and Mechanical Cues

Different microstructure and surface morphology such as amorphous, porous, composite structure significantly influence the corrosion performance of Mg alloys. Amorphous Mg_66_Zn_30_Ca_4_ exhibits superior corrosion resistance compared to the crystalline Mg_60_Zn_35_Ca_5_ (Ramya et al., 2015). In addition, the partially crystallized metastable structure of Mg_108.08_Zn_39.6_ exhibits best corrosion resistance in SBF solution when compared to crystalline structure (Wang et al., 2012). A porous structure of the alloy is ideal as it mimics the structure of spongy bone for improving corrosion resistance and scaffold applications in which case Mg scaffold degrades fast but promotes the bone remodeling process with good biocompatibility. The composite material in combination with pure Mg and 20 wt% of ZnO powder exhibited improved mechanical strength with good corrosion resistance compared to pure Mg in Hank's solution (Lei et al., 2012). Surface morphology could also be tailored by grafting approach by introducing brushes or coating. For bioinert surfaces or coating, the surface chemistry and topography, can affect the extent of tissue bonding to the implant. On this basis, surfaces can be classified as either bioactive or bioinert, depending on the host response elicited when implanted in skeletal tissues.

#### Micro-Nanotopography of Mg Alloy

**Figure 4:**
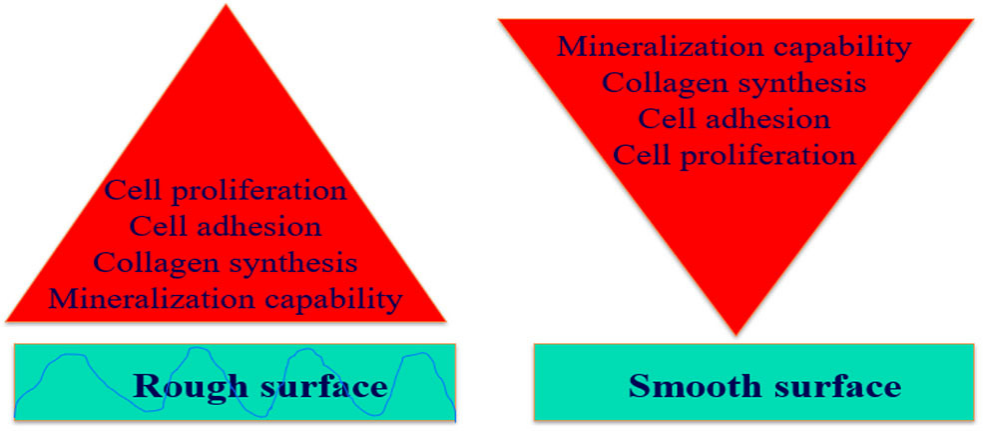
Effect of surface roughness on cellular behavior.

Physical environment such as surface roughness of the metal implant influences the cell adhesion, recruitment and migration. Response of osteoblast to surface structure reveals that the grooved surfaces can guide cell migration. In general, rough surface provides more protection against corrosion along with better bone integration compared to smooth surface through new bone generation because of the more exposed area. Interaction between implants surfaces and tissues is ideally controlled by the texture of implant surfaces with better tissue growth on the micro-textured surface than very polished surface (Lacefield, 1999; Parekh et al., 2012). Fabrication of anodized surface of ZK60, AZ91E, and AZ31B Mg alloys shows corrosion performance and cellular response in an artificial body fluid environment with AZ91E Mg alloy exhibiting highest surface roughness amongst other Mg alloys and offered best corrosion protection ability with best biocompatibility due to unique micronanotopography. Additionally, microroughness can affect the progression of osteoblast phenotype by upregulating integrins such as α2β1, which subsequently regulates osteoblast differentiation and local factor production. Substantially higher rates of collagen synthesis and mineralization capability for cells are expected on rough surface than on smooth surfaces (Figure 4). In general, the adhesion strength and cell adhesion decrease at the smooth surface, the bonding strength and cell proliferation increase with increase in surface roughness as shown in Figure 4.

## Tuning Interface Between Mg Alloys and Coatings for Corrosion Control

**Figure 5:**
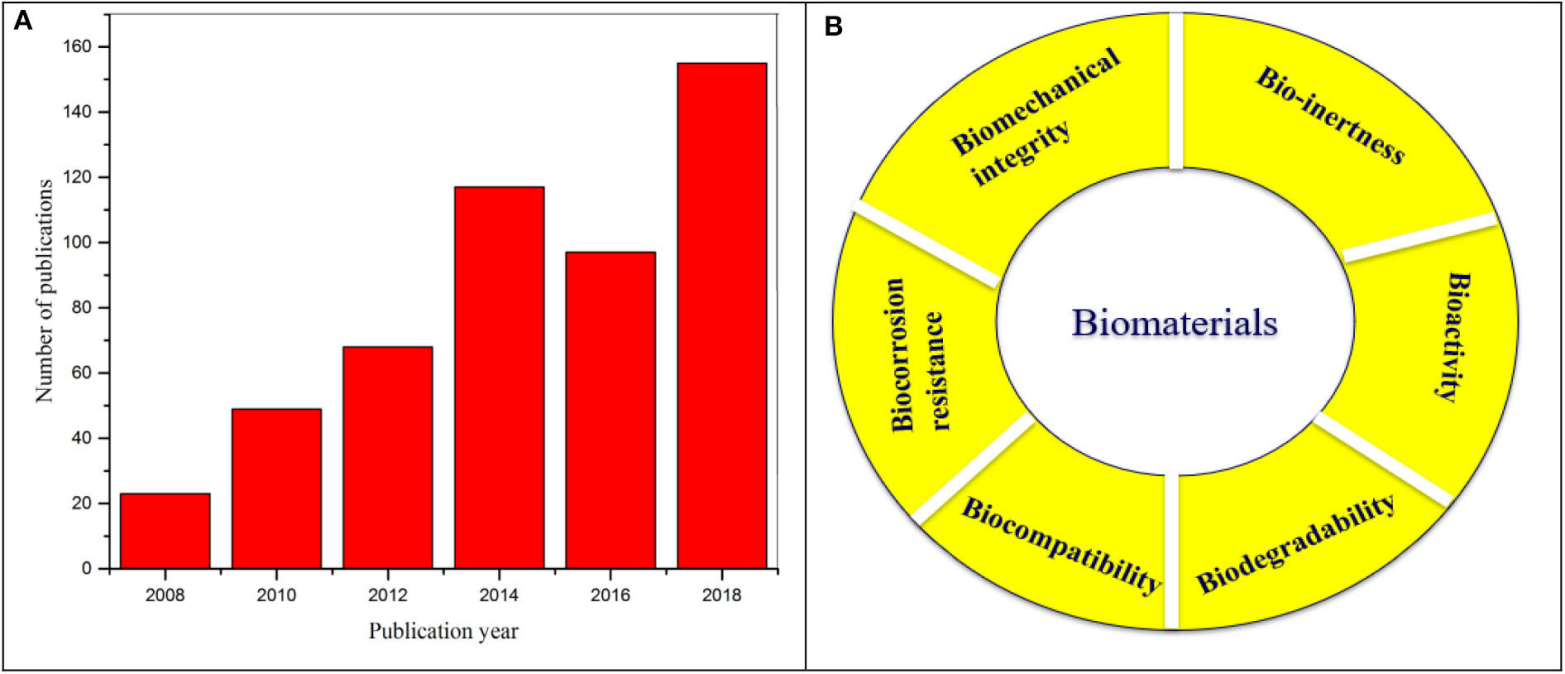
**(A)** Illustration of number of scientific publications from 2008 to 2018 using the search terms “magnesium” and “biomedical applications”. Data analysis was performed on 14 August 2019 using Scopus search system. **(B)** Basic requirements of biomaterials.

Research on Mg alloys for biomedical applications have grown significantly over last decade and the scientific publications in the area demonstrate the impact of this field with respect to various strategies of bio-interface control as shown in Figure 5A. While surface coating offers an avenue to improve corrosion resistance and cytocompatibility of Mg alloys, however, often water permeability and swelling of the coating could initiate corrosion of the Mg substrates. In order to improve the interface strength of the coating and the substrate, a key feature is to combine surface modification and surface/polymer coating on the substrate. This section highlights the different bio-interface modification techniques and recent development and challenges of Mg alloys. The design and requirement of properties for interface modification of Mg alloy are many and including bioactivity, biocompatibility, controlled degradability, mechanical integrity, osteo-conductivity as shown in Figure 5B.

### Micro/Nano Structure Fabrication on Mg Substrates

#### Chemical Surface Modification of Mg Alloys-Fluoride Pretreatment

Fluoride based conversion coating has received significant attention due to its simplicity and affordability. Fluoride treated Mg alloy substrate using hydrofluoric acid enhances corrosion resistance, antibacterial properties and improve osseointegration as compared to untreated Mg alloys (Berglundh et al., 2007). The concentration of the solution and treatment time dominate the coating thickness and corrosion properties of Mg-based alloys. Fluoride- coating fabricated on AZ31 Mg alloys using 50% fluoride solution shows significant improvement of corrosion with the corrosion value shifting to nobler direction with treatment time. The corrosion performance does not show any dependence on concentrations of fluoride solution (Da Conceicao et al., 2010). However, the highest coating thickness and best uniform coating layer was obtained with 40% HF treated substrate (Bakhsheshi-Rad et al., 2013). In general, the treatment time is directly proportional to coating thickness, rapid corrosion and initial degradation can be delayed with thick coating (Fintová et al., 2019). Fluoride-based treatment not only improves biocompatibility but also offers appropriate protection against corrosion with the formation of MgF_2_ and improved adhesion strength confirmed by scratch tests due to the formation of barrier type film (Drábiková et al., 2018). Fluoride/biopolymer coated substrate has shown enhanced corrosion resistance along with increased cell adhesion, cell viability, and cell proliferation on the surface of the substrate (Lee et al., 2019). Also with fluorinated HA (FHA), Mg-Zn-Zr-Sr Mg alloy shows superior corrosion resistance and good biomineralization ability as compared to untreated Mg alloy (Razavi et al., 2015). However, a very few studies have been reported on fluoride-pretreatment coupled with various surface coating and such coating not only improves the wear resistance but also adhesion strength between substrate and coating (Tran et al., 2006). In summary, an alkali treatment or acid treatment is an approach of nanostructured surface modification which delays the initial corrosion and degradation of Mg substrates in the physiological environment by forming a barrier type film layer on the surface of the substrate.

#### Ceramic Coating on Mg Alloys

Ceramic materials have long been studied as potential candidate for bone tissue engineering. HA Ca_10_(PO_4_)_6_(OH_2_) is one of the major inorganic compounds of bone tissues and has been successfully used in biomedical applications specially in dentistry. Basically, there are different phases of CaP ceramic including HA, tricalcium phosphate (TCP), dicalcium phosphate dihydrate (DCPD), bi-phasic calcium phosphate (BCP). Actually, CaP crystals are designated depending on the molar ratio of Ca/P such as HA, DCPD, TCP are 1.67, 1 and 1.5, respectively (Dorozhkin, 2014). These coatings are considered to improve biocompatibility, bioactivity, osteo-conductivity, corrosion resistance, and control degradation behavior of Mg-based implant materials (Liao et al., 2013). HA is one of the most potential phases of all CaP due to its chemical and natural bone mimic structure and can improve bone regeneration (Burg et al., 2000). However, HA coating can be synthesized by different techniques including anodization, cathodic deposition, biomimetic process, and electrophoretic deposition. Anodization is a simple electrolytic process (Blawert et al., 2006) for fabricating a dense protective oxide layer on the surface of the Mg and its alloys that can improve corrosion resistance. Mousa et al. (2015) fabricated a nano-structured HA coating on AZ31B combining anodization/hydrothermal processes, that exhibits higher corrosion resistance and increased bioactivity and biocompatibility than the uncoated one. HA exhibits strong propensity for attracting osteoblast but possesses a low reabsorption rate *in vivo* and brittle when specially in porous form. The anodized layer can give extra protection against corrosion while HA based bioactive coating can be fabricated on the surface of the substrate, enhancing osteoconductive properties of Mg alloys. An alkali treatment with HA coating on Mg alloy can significantly improve corrosion resistance and enhance biocompatibility and bioactivity due to the double layer on the substrate (Song et al., 2008). The biocompatibility and bioactivity of HA coatings are two exclusive properties over other coatings which make them potential incumbent as an anticorrosion coating for bioresorbable and biodegradable Mg alloys. It offers controllable corrosion properties to protect the substrate against corrosion at the early stage of implantation and enhances the osseointegration ability (Dunne et al., 2015).

Different strategies such as electrophoretic process, sol-gel derived dip coating methods, electrodeposition method, anodization process can be used to fabricate nanostructured HA coating on Mg alloys. The HA-coated Mg-3Zn alloy shows superior corrosion resistance -about 25 times more than that of uncoated Mg alloy, as confirmed by *in vitro* assessment (Kumar et al., 2016). Moreover, mechanical properties of the metal such as hardness, elastic modulus, and fracture toughness increase as compared to bare Mg alloy substrate. A nanostructured HA coating on Mg-3Zn alloy prepared by electrophoretic deposition process shows significantly enhanced cell viability and adhesion strength (Sankar et al., 2017). A nanostructured HA coating fabricated on Mg substrate using dip coating method followed by a chemical treatment, on the other hand, gives adequate resistance to the additional release of Mg^2+^ thus, improves corrosion resistance and biomineralization through stabilizing the alkalization characteristics (Rojaee et al., 2013).

### Polymer Coatings on Mg Alloy Surface

**Figure 6:**
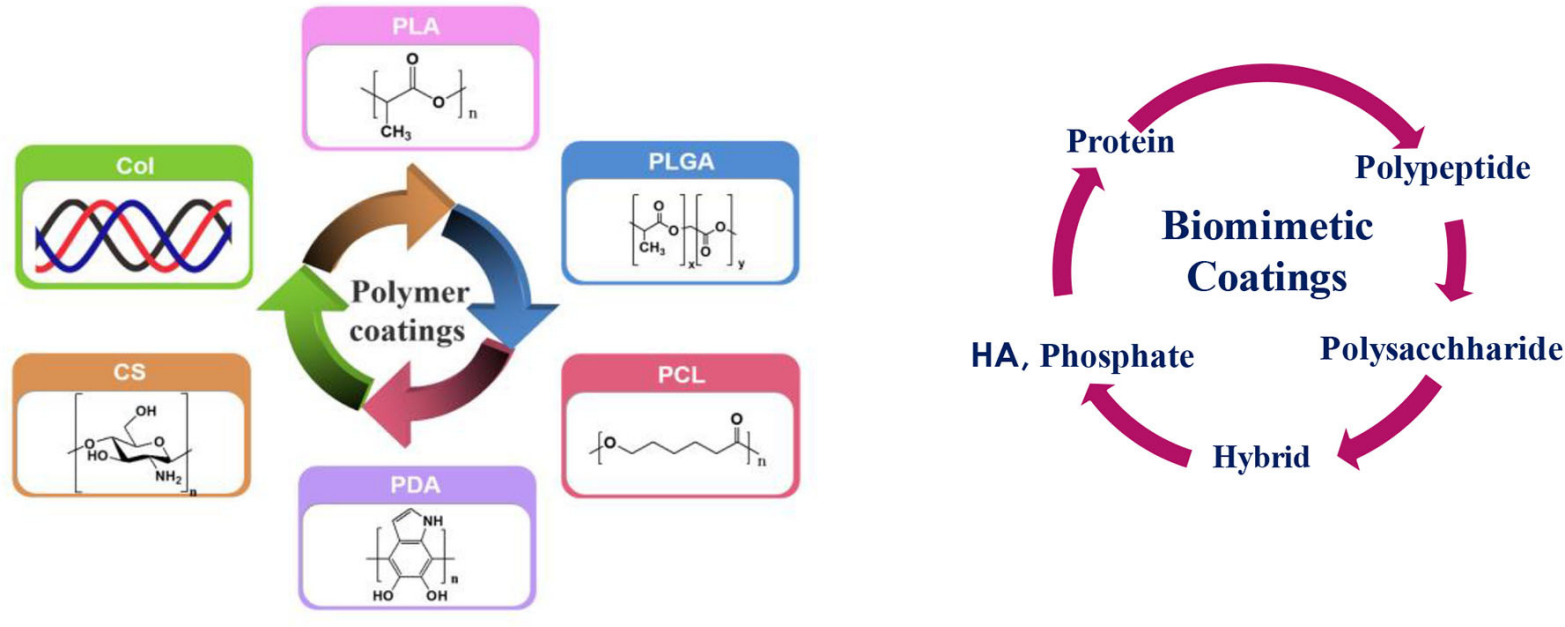
Schematic illustration of various coatings: **(Left)** polymer coatings: Col, CS—bioactive coatings, PLA, PLGA, PCL, PDA-bioinert coatings and **(Right)** biomimetic coatings (**Left** figure: Reprinted with the permission; Li et al., 2018). Copyright 2018, Elsevier.

Although HA has strong osteo-conduction property, but its low resorption rate *in vivo* and brittleness and porous form pose problem. These challenges could be overcome by combining HA with organic polymers. Hybridization of such organic and inorganic components is an important and evolutionary route for the growth of strong organic-inorganic interface. This could be done either by combining them in one composition as hybrid coating or applying them in layer by layer fashion. The resulting nanostructure, degree of organization, and the properties achieved primarily depend on the chemical nature of the components used, the nature of the interface that controls the synergy between the two components. Amongst various polymers, biodegradable polymers such as polyglycolic acid (PGA), polylactic acid (PLA), polycaprolactone (PCL), and polyhdroxubutyrate (PHB) (Peppas and Langer, 1994) are best candidates for making such coatings that are reported in the literature. A schematic illustration of such potential coatings is presented in Figure 6 and will be discussed in the following section.

#### Bioinert and Biodegradable Coating

**Figure 7:**
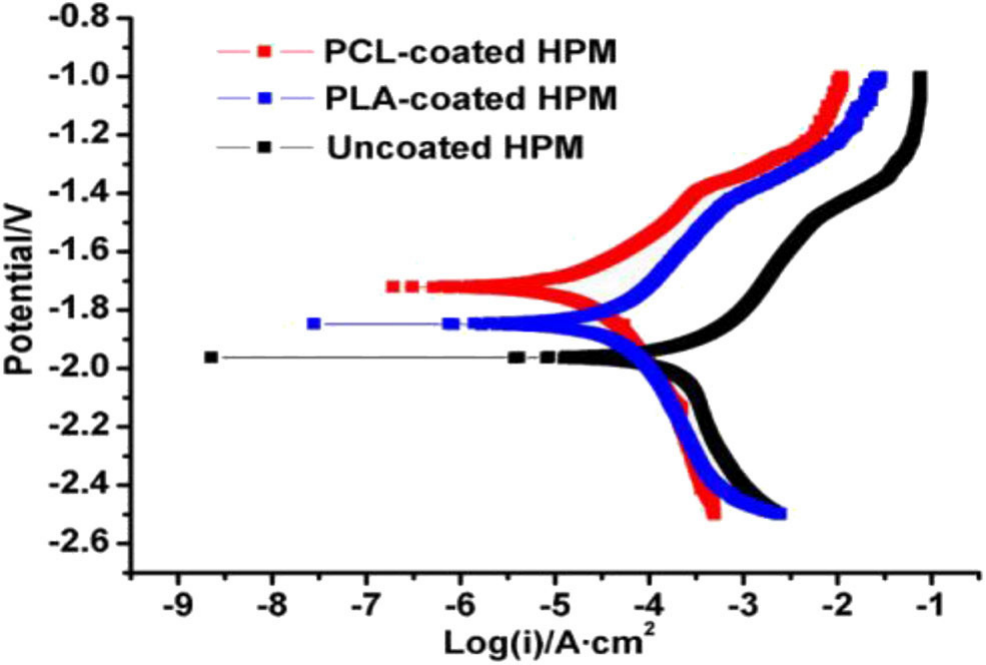
Potentiodynamic polarization curves of PCL, PLA, and uncoated AZ91 Mg alloy in SBF solution. Reprinted with permission (Chen et al., 2011). Copyright 2011, IOP publishing.

Bioinert coating on metal offers a potential strategy to modify or protect the surface properties of implantable device using biocompatible polymer coatings capable of limiting non-specific protein adhesion and attachments**. **The bioinert, biodegradable and bioresorbable polymers are PLA, PLLA, PLGA, PCL which are widely used for biomedical applications. The bioinert and bioresorbable polymer should gradually degrade in the physiological environment without producing any adverse effect. So far, many experiments have been performed to assess the corrosion and degradation of bioresorbable polymers including PLA and PCL for clinical applications (Schliecker et al., 2004; Lyu et al., 2007; Gleadall et al., 2014). However, PLA and PLGA produce non-toxic degradation products in the physiological environment due to their biocompatibility which made them potential candidates for many pharmaceutical and medical applications (Alsaheb et al., 2015). PLLA coatings exhibited better adhesion strength to Mg substrate compared to semi-crystalline PCL layers and improved the corrosion resistance and cytocompatibility of Mg substrate (Xu and Yamamoto, 2012). In contrast, the major limitation of the PLA is poor mechanical integrity compared to natural bone as a load bearing implant material for orthopedic applications (Wang et al., 2016). PCL has attracted much attention in biomedical applications due to its good biodegradability, excellent biocompatibility, easy handling, inexpensive manufacturing process and well-control over the corrosion rate (Mondal et al., 2016). By comparison with other polymers, the rate of degradation of PCL is very slow and hence, it limits some specific biomedical applications and to overcome such limitations necessitates modification such as making composite materials or coatings with synthetic or natural polymers (Abedalwafa et al., 2013). Chen et al. (2011) demonstrated PLA and PCL coatings on high purity Mg (HPM) sample to improve corrosion resistance and they reported that the polymer coated Mg substrate significantly improved corrosion resistance compared to uncoated substrate (shown in Figure 7). Immersion tests also confirmed that the PCL and PLA coated samples reduced the degradation rate in SBF solution.

Both PCL and PLA/PLGA polymer coating have been found biocompatible and biodegradable with metabolically digestible degradation residues and hence recently got approval from US Food and Drug Administration (FDA) for medical devices (Brown et al., 2015). Moreover, the homopolymer PCL is referred to as anticorrosion coating as well as beneficial for drug delivery and slowly degrades and offers resistance for coating dissolution in biofluids compared to that of PLA (Yazdimamaghani et al., 2015). The main limitations of PLLA coating are poor mechanical properties because of the high affinity to hydrolysis in water as compared to natural bone that makes it unsuitable as load bearing applications causing implant failure (Agarwal et al., 2016). Considering this limitation, a composite coating can be adopted to make a promising coating for Mg implant. However, polymer that can offer controllable corrosion rate in long term degradation still is a challenge. A bilayer composite coating including inner layer with PLLA and outer layer with DCPD was fabricated on Mg alloy with a pretreatment of UV irradiation to accelerate heterogeneous nucleation of the DCPD coating for enhancing biocorrosion resistance, biocompatibility, and osteocompatibility and cellular response in terms of cell adhesion, cell viability, and ALP assay tests, respectively (Zhang et al., 2017). Collagen (Col) and Chondroitin sulfate (CS) are bioactive coatings that have been used for implant coating (Li et al., 2018).

#### Biomimetic Coating

A paradigm shift in the design of new biomimetic coating from inert biomaterials is to the utilization of structural materials derived from extracellular matrix protein. The existence of biomolecules such as protein, peptides, polysaccharides (Figure 6) has significant effect in modifying cytocompatibility of biodegradable polymers. Due to their inherent structure and composition, they create bioactive or biomimetic materials that elicit controlled adhesion, osteo-conductivity, and stability of the bone implant interface. Lin et al. (2015) synthesized dopamine induced biomimetic HA coatings on AZ31 and reported that the coated substrate is homogeneous and uniform with better L-929 cell adhesion and proliferation as confirmed by the cytotoxicity testing.

Collagen and chitosan coatings are considered as natural polymer coatings. Collagen is one of the prime components of natural bone matrix. It serves mostly mechanical purposes and constitutes the organic matrix of bone and dentin as well as the bulk of muscles and ligaments. It is anticipated that a collagen coating on Mg alloys may be an appropriate strategy to suppress the corrosion in the biological environment and improve osseointegration properties. Natural biopolymer like chitosan dominates the numerous biological activities such as controlling the degradation rate, corrosion rate and enhances mechanical properties and osteo-conductivity. HA-chitosan composite coatings using electrophoretic or electrochemical deposition process are beneficial to improve the mechanical integrity and biological properties including bioactivity, biocompatibility, and osteo-conductivity (Redepenning et al., 2003; Pang and Zhitomirsky, 2007).

Biomimetic coating combining HA and collagen significantly improves corrosion resistance compared to HA-coated and uncoated substrates due to their chemical stability and less dissolution of Mg^2+^ ions (Bao et al., 2017). However, HA/collagen coated AZ31 Mg alloy sample shows excellent chemical bonding between HA/collagen like natural bone and suppresses the sudden release of Mg^2+^ ions and with pH value increment of the Hank's solution the degradation rate is reduced with improved corrosion resistance (Wang et al., 2013). The most prominent quality of collagen polymer is the capability to self-assemble into fibrils, results in a classified tissue structure. The structural hierarchy caused by the collagen assembly offers the mechanical toughness and resilience required for the tissues to perform efficiently in the biological environment (Boskey et al., 1999). Chitosan, a natural polymer, non-toxic, and biocompatible polymer consists of N-acetylglucosamine and glucosamine units combined by one to four glycosidic bonds (Delezuk et al., 2017), which makes it fascinating for use in bone-tissue engineering, drug release carrier and artificial skin (Ordikhani et al., 2014). The chitosan coatings were prepared on porous Mg with different molecular weight such as 1.0 × 10^4^, 1.5 × 10^5^, 2.7 × 10^5^, 6.0 × 10^5^ but the coated substrate with molecular weight of 1.5 × 10^5^ and 2.7 × 10^5^ showed very smooth surface whereas 1.0 × 10^4^ and 6.0 × 10^5^ molecular weight surfaces exhibited some cracks and holes on the surface of the substrate (Gu et al., 2009). The immersion test reveals that the chitosan with highest molecular weight of 2.7 × 10^5^ exhibits best corrosion resistance in terms of volume of H_2_ gas evolution and pH value of the medium as compared to other chitosan due to uniform, compact, and thicker coating layer. In addition, corrosion resistance depends on the porosity percentage of Mg alloys. The effect of porous Mg monoliths with different porosities of 14, 30, 40 vol% has been studied and the highly porous Mg with chitosan coating quickly penetrated and converted into monolith structure which results in uneven surface (Tiyyagura et al., 2016). Thus, chitosan coated highly porous Mg monoliths make a barrier between the substrate and the body fluid solution leading to excellent corrosion resistance.

Silk fibroin (SF) is a fibrous protein based natural polymer with potential properties for biomedical applications. The natural protein SF is extracted from *Bombyx mori* silkworm. So far, very few works have been reported with SF on Mg alloys for clinical applications. Hence, there is a great opportunity to work with this promising material to enhance the biological therapies. Due to its hydrophobic nature, SF has the ability to improve corrosion and degradation properties, mechanical properties, and biocompatibility (Rockwood et al., 2011) and such fibrous coating material could be potential means to overcome the limitations of using Mg based alloys. Also, due to its superior corrosion resistance (Wang et al., 2008), adjustable biodegradability (Kundu et al., 2014; You et al., 2014), and excellent biocompatibility (Thurber et al., 2015), silk fibroin exhibits incredible potential for coating applications in comparison with other polymeric coatings including certain composite bio-coatings (Xiong et al., 2017), apatite (Jiang et al., 2018), and ceramic (Rau et al., 2016). While synthetic polymers such as PLA, PLGA, and polyacrylic acid (PAA) release acidic products and increase foreign body response during corrosion and degradation of implants, in contrast, SF is safer to that of bioinert and other biomimetic polymers (Xiong et al., 2019).

### Bioactive Hybrid Coating

The term hybrid coating can be produced in combination of organic and inorganic substances. Inorganic coatings include chemical conversion coating (alkali treatment, hydroxyapatite coating, calcium phosphate coating, ion implantation etc.), ceramic coatings (titania, silica, alumina, zirconia), carbon, glass and so on (Van Delinder and Brasunas, 1984). Organic coatings consist of epoxy, polyurethane, poly(methyl methacrylate), fluoropolymers, polyesters, silk fibroin as well as allied paints, associated with different types of pigments, are extensively applied as protective coating. There are different ways to develop a hybrid coating including epoxy-silane coating, combination of both inorganic and organic coatings, and layer by layer coatings discussed in the following sections.

#### Nanocomposite Hybrid Coatings

**Figure 8:**
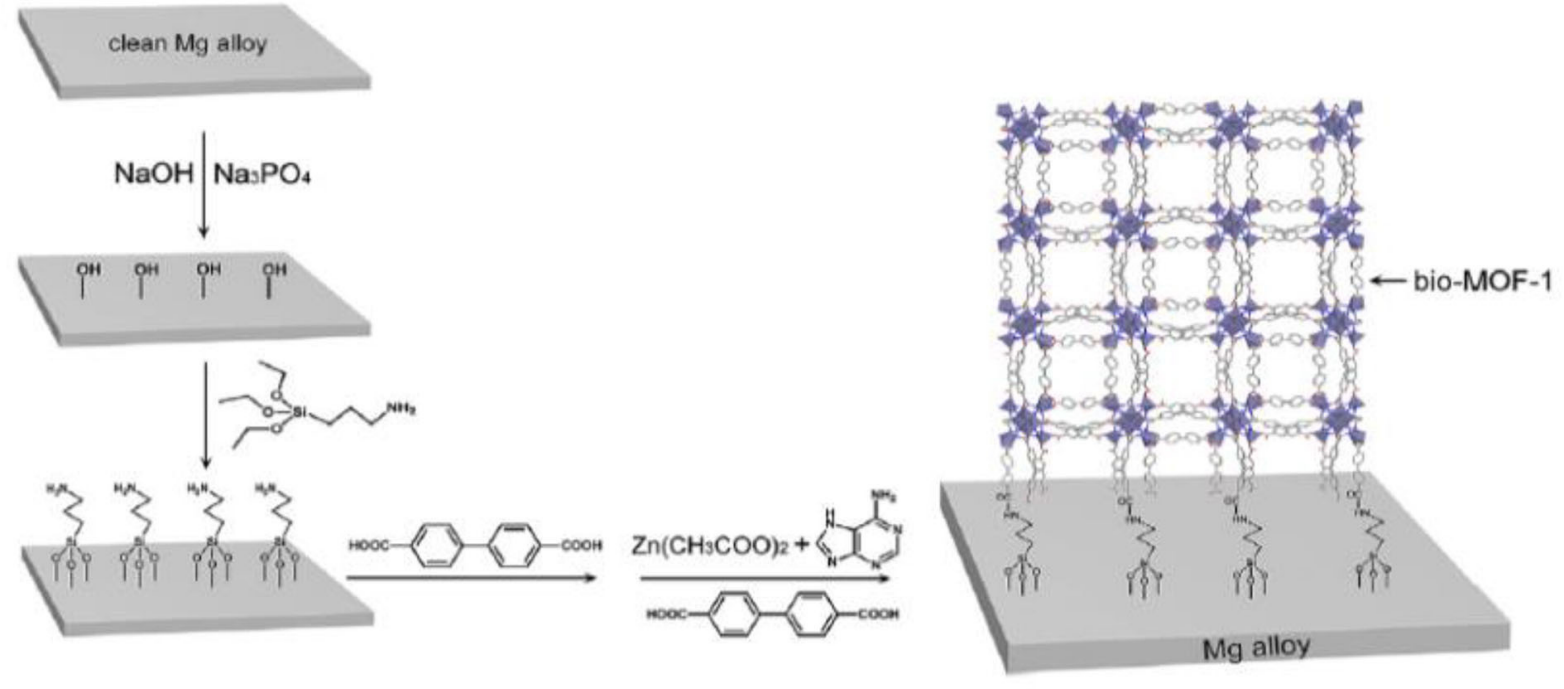
Schematic structural formula of Bio-MOF coating on Mg alloy. Reprinted with permission (Liu et al., 2019). Copyright 2019, Elsevier.

Composite coating on Mg and its alloys is one of the recent developments to suppress the corrosion and degradation rate in the biological environment. The corrosion resistance of AZ31 significantly improved with high adhesion strength (24.6–27.7 MPa) as compared to bare substrate (Hahn et al., 2011). Besides, Bai et al. (2012) prepared a composite coating combining chitosan/MAO on Mg-Zn-Ca sample to evaluate electrochemical performance. The anodic polarization measurements reveal that the composite coated Mg alloy significantly enhances the corrosion resistance thus slows down the degradation rate in SBF solution as compared to untreated Mg alloy substrate. A composite coating consisting of hydroxyapatite compounds, various diethylene triamine, and polyetherimide fabricated on pretreated AZ31 Mg alloy (Zomorodian et al., 2013), greatly enhanced its corrosion resistance compared to the uncoated sample. However, as bone implant coating, metal organic frameworks (MOFs) are also promising candidates for surface functionalization. Their high specific surface area can be beneficial for cell adhesion and proliferation; their pore structures and pore volume offer great potential for adding and rejecting some biological molecules or antibacterial agents. Such diverse functional groups provide different chemical characters to Mg alloy implants. Liu et al. (2018) fabricated a Mg-MOF-74/MgF_2_ nanocomposite coating (Figure 8) that greatly enhanced corrosion resistance, slowed down degradation rate, and increased hydrophilicity of composite coated substrate. MOF-1 coated on AZ31 Mg alloy substrate not only enhanced corrosion resistance but also demonstrated excellent bioactivity as compared to uncoated Mg alloy (Liu et al., 2019).

#### Layer by Layer Coatings

The interfacial engineering approach with bio-macromolecular layer by layer (LBL) coating can be designed to increase the stability of coating as compared to single layer coating and for controlled delivery of bioactive molecules or growth factors. In SBF solution, a single layer treated Mg sample surface might not satisfy the requirements as implant materials (Wu and Zhang, 2011). Thus, LBL coating not only strengthens implant life but also offers tailorable surface properties of the coating in terms of microstructure, coating thickness, and mechanical properties (Decher, 1997). A layer by layer coating on AZ31 Mg alloy using a novel spin coating technique shows enhanced corrosion protection ability due to crack free and compact coated surface (Zhao et al., 2018). For Mg substrates, hybrid coating by LBL is an emerging research area for biomedical applications. Yang et al. (2018) produced a hybrid plasma electrolytic oxidation (PEO) coating on anodized Mg using dip coating method. The authors reported that the inner layer is very compact between two distinctive layers, but the immersion repetition time dominated the thickness of the coating. The outer layer of the coating is porous made of PEO coating which exhibits some cracks and micropores, in contrast, the inner layer is defect free and compact. Therefore, such a promising coating technique can tune surface properties of Mg-based implant materials for biomedical applications. Patil et al. (2017) synthesized hybrid self-assembled multilayer anticorrosion coatings on Mg sample using dip coating method and observed homogeneous, crack free, and micron thick coating layer. The layer with alkali treated multilayer coating exhibited best corrosion resistance in terms of volume of H_2_ evolution, weight loss measurements, and potentiodynamic polarization results after immersion of 7d in SBF solution.

A comparison of different coating's performance is shown in Table 2 with respect to different corrosion medium. Hybrid coating offers double benefit as compared to single layer of inorganic or organic coating. A functional hybrid coating was fabricated on biodegradable AZ31 and ZE41 Mg alloys which consists of inner layer of the Mg implants made of silane-TiO_2_ to reduce degradation rate and the topmost layer made of biopolymer composed of chitosan and collagen to improve biocompatibility and bioactivity of implant materials (Córdoba et al., 2018). No unfavorable effect was observed on the barrier type silane-TiO_2_ coated layer whereas the top layer resisted the formation of gas pockets for deposition of H_2_ gas. EIS results confirmed the highest corrosion resistance for long-term protection after immersion in SBF solution for 7d due to barrier type coatings on Mg alloys. Hybrid epoxy-silane coatings on Mg alloy show enhanced corrosion protection ability as compared to the uncoated specimen as evident from both immersion and EIS tests in 0.5 NaCl solution due to multiple layers on the substrate (Brusciotti et al., 2013).

**Table 2:** Corrosion performance of different coating processes.

**Substrate**	**Coating**	**Corrosion medium**	**Corrosion voltage (V)**	**Corrosion current (μA/cm^**2**^)**	**Corrosion resistance (kΩ cm^**2**^)**	**References**
Mg-Zn-Ca	HA	SBF	−1.41	25	–	Wang et al., 2010
AZ31	HA	SBF	−1.57	5.56	–	Sun et al., 2019
AZ91	Hybrid	3.5% NaCl	–	–	430	Ashassi-Sorkhabi et al., 2019
AZ31	Composite	SBF	−1.62	3.4 × 10^−4^	–	Wei et al., 2014
AZ31	PLA	SBF	−1.57	7.72	–	Shi et al., 2015
Mg-6Zn	PLGA	0.9 NaCl	−1.44	0.085	–	Li et al., 2010
Mg	PCL	Hank's solution	−1.53	0.0045	–	Li et al., 2014
AZ31B	Hybrid	0.005 NaCl	–	–	160	Lamaka et al., 2008

## Tuning Interfaces Between Mg Alloys for Controlling Cellular Environment

### Osteointegration of Coatings

Osseointegration (OI), the functional connection between bone and implants, is a pivotal process for implant fixation and integration. In the biological media, the implant system is in a microenvironment of three distinct phases: solid implant (macroscale), solution phase (macroscale), and solid-liquid interface (nanoscale). The initial interaction of the Mg surface with the biological environment is based on the adsorption of proteins on its surface and interaction with ions and water molecules. Depending on surface physico-chemical properties and their interactions, proteins adsorb on different substrate in differing quantities, densities, conformations, and orientations. The attachment or focal contact occurs rapidly with short-term physicochemical linkages between cells and materials involving ionic forces, van der Walls forces, etc. followed by the adhesion phase involving various biological molecules: extracellular matrix proteins, cell membrane proteins, and cytoskeleton proteins which interact together to induce late events, related to cell proliferation, differentiation and cell function through signal transduction, transcription factors, and regulation of gene expression. Understanding the complex physical and chemical interactions that take place at the interface between the Mg surface and bone tissue is crucial for controlling their integration. Bone is a 3-dimensional inhomogeneous structure with complicated topography. If the Mg implant matches similar hierarchical structure of bone in multiple length scales, then OI is facilitated.

During osteointegration, cells at the interface and their secreted proteins alter the structure and physico-chemical properties of the implant surface. Continuous electrochemical events take place at the tissue-implant interface such as metal ions released from the implant into tissue; these ions can be traced in the peri-implant tissues or other organs. However, excessive metal ion release can inhibit cell function and apatite formation. Use of various surface characterization approaches enable evaluation of implant performance through coating-tissue integration or through migration of metal ion from implant to tissue. Histological methods, interfacial mechanical property evaluation through nanoindentation, structural information through SAXS, Raman microscopy, and elemental analysis enable characterization of the quality of the interface, bone formation, mechanical, structural, and adhesion analysis. The interfacial bonding between the surrounding bone and the implant surface thus plays crucial role in dictating implant failure (Bobyn et al., 1995; Urban et al., 1996; Patel and Gohil, 2012). Traditional biodegradable polymers (e.g., PLGA, PLA, and PCL) frequently suffer from comparatively poor adhesion to Mg based implants, thus initiating interfacial failure at early stage of implantation (Xu and Yamamoto, 2012; Kim et al., 2013). To resolve or improve the coating stability, poly(ether imide) (PEI) polymers have been recommended as an alternative coating material because of its outstanding adhesion strength to Mg, caused by its polar aromatic imide rings, moreover its good biocompatibility and flexibility (Abdal-hay et al., 2013).

**Figure 9:**
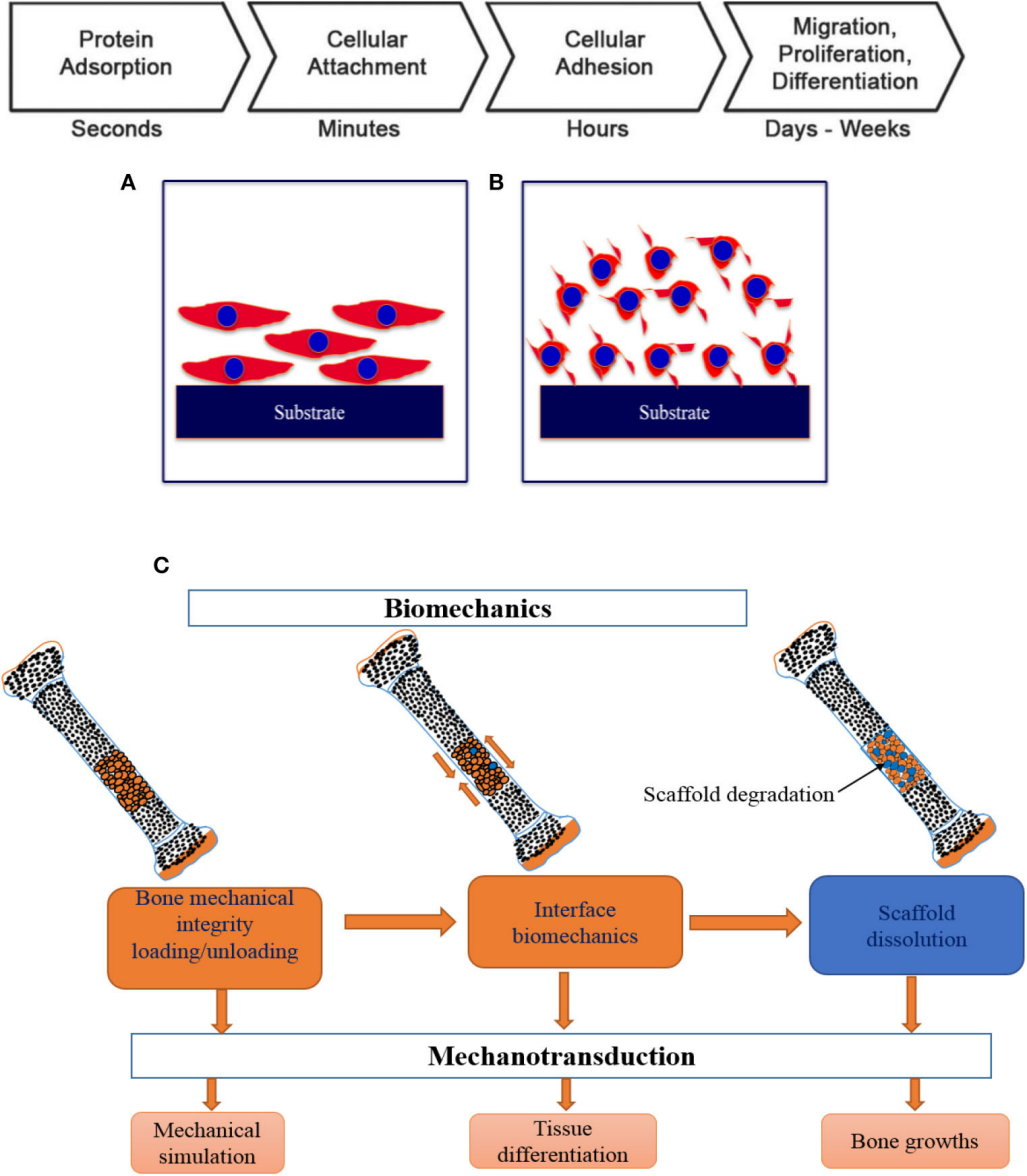
Schematic of cellular response in different environment in various timescale: **(A)** osteoblast cells with physico-chemical interaction, **(B)** cell attachment with integrin binding, and **(C)** key steps in osseointegration and bone TE. Adapted with permission from Pioletti (2010).

**Figure 10:**
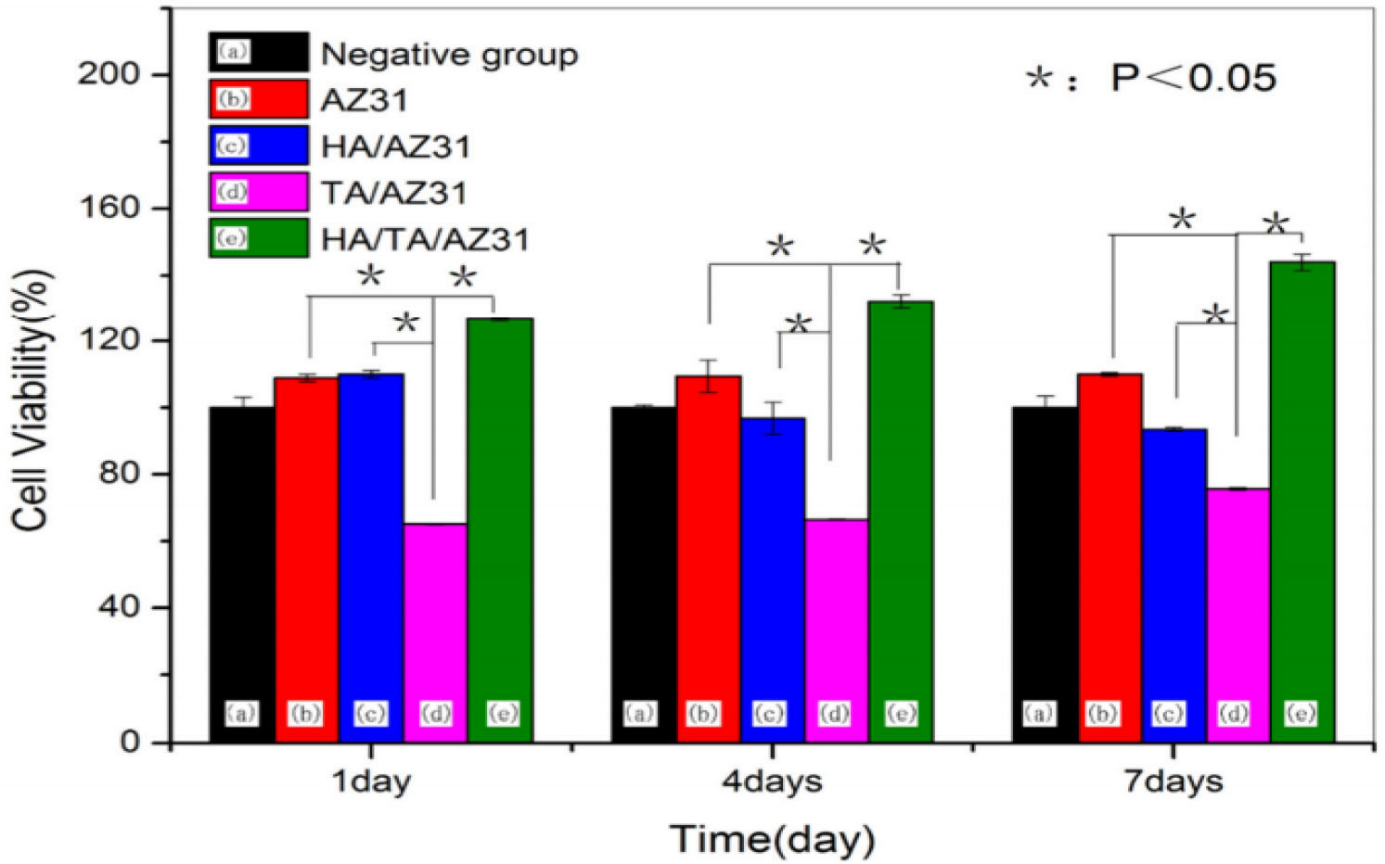
Cell viability of uncoated and hybrid coated AZ31 Mg alloy for different immersion periods. Reprinted with permission (Zhu et al., 2017). Copyright 2017, MDPI.

The very fundamental cellular behavior including cell migration, adhesion, proliferation and differentiation is greatly influenced by the native environment and extracellular matrix (ECM) as the ECM offers adequate mechanical integrity for cells as is shown in Figure 9 with a schematic of the cell attachment and proliferation process with a time scale. The cells obtain appropriate cues on physical and chemical nature of the environment while directly interact with ECM and then interpreting and integrating them, generate proper physiological response (Spatz and Geiger, 2007). So, the appropriate and convenient environment is very important for such superb sensitivity and intelligence of the cells and the cellular behavior can also be modified by precisely designing the environment. For cell migration, the contact guidance that guides the direction of cell alignment and enlargement of the cells on the surface of the substrate is crucial. For example, the level of alignment such as depth and width of groove of the surface of the implant dominates the cell migration as the cells proliferate along the grove direction and depth of the groove (25 μm) of topographies (1 μm) although it varies depending on the cell lines (Wilkinson et al., 2002). On the other hand, a study with fibroblast cell on the polystyrene substrate with nano-grooved (depth 5–350 nm and width 20–1,000 nm) was performed to understand the performance of contact guidance on topographies. The fibroblasts limit their alignment on the surface of substrate in the range of depth below 35 nm and width lower than 100 nm of the groove (Loesberg et al., 2007). Moreover, it was reported that the fibroblasts and mesenchymal cells prompt distinct ability of adhesion and proliferation on the nanoscale surface patterns (heights <10 nm), although both cells significantly adhered and proliferated on the coated substrate (Khor et al., 2007). The cells start migrating to colonize around the implant surface at the first few days of implantation to increase mineralization and extracellular matrix although healing process starts through an inflammatory response. The coated rough surface is more effective for proper osseointegration compared to polished implant surface. In case of Mg implant materials, the release of Mg ions increased osteogenic signals that can lead to rapid bone growth spread on the implants surface. The hybrid coating was fabricated combining HA/tannic acid (TA) on AZ31 Mg alloy to improve cell adhesion and proliferation (Zhu et al., 2017). In Figure 10, the MC3T3-E1 cell viability gradually increased with increase in the cell culture time which indicates HA/TA coating effectively offers protection against early corrosion in the cell culture media. The cell morphology and cell proliferation observation indicates that HA/TA coated substrate is non-toxic to the MC3T3-E1 cells with the cell viability ~100%.

In general, the adhesion strength is very important factor either in long term implantation or in surgery process in the physiological environment. In case of polymeric substrate, the moderate wettability (contact angle 40–60°) enhances the cell attachment capability around the affected surface (Arima and Iwata, 2007). It is reported that cell attachment is not only dominated by surface wettability but also surface functional groups, surface roughness, their surface density as well-types and natures of the cells. On the other hand, the metallic substrate having lower contact angle below 30° are most promising for effective osseointegration. However, rough surface enhances the cell attachment ability as the rough surface provides extra anchorage as compared to smooth surface. Overall, the new bone tissue growth can progress from the host bone tissue to the surface of the implant, thus, gradually covering up the gap, which results in device integration. While the macroscale aspects of the implant are important, intrinsic surface characteristics at the micro-, submicro-, and nanoscale must be considered simultaneously to ensure the ability of osteointegration.

**Figure 11:**
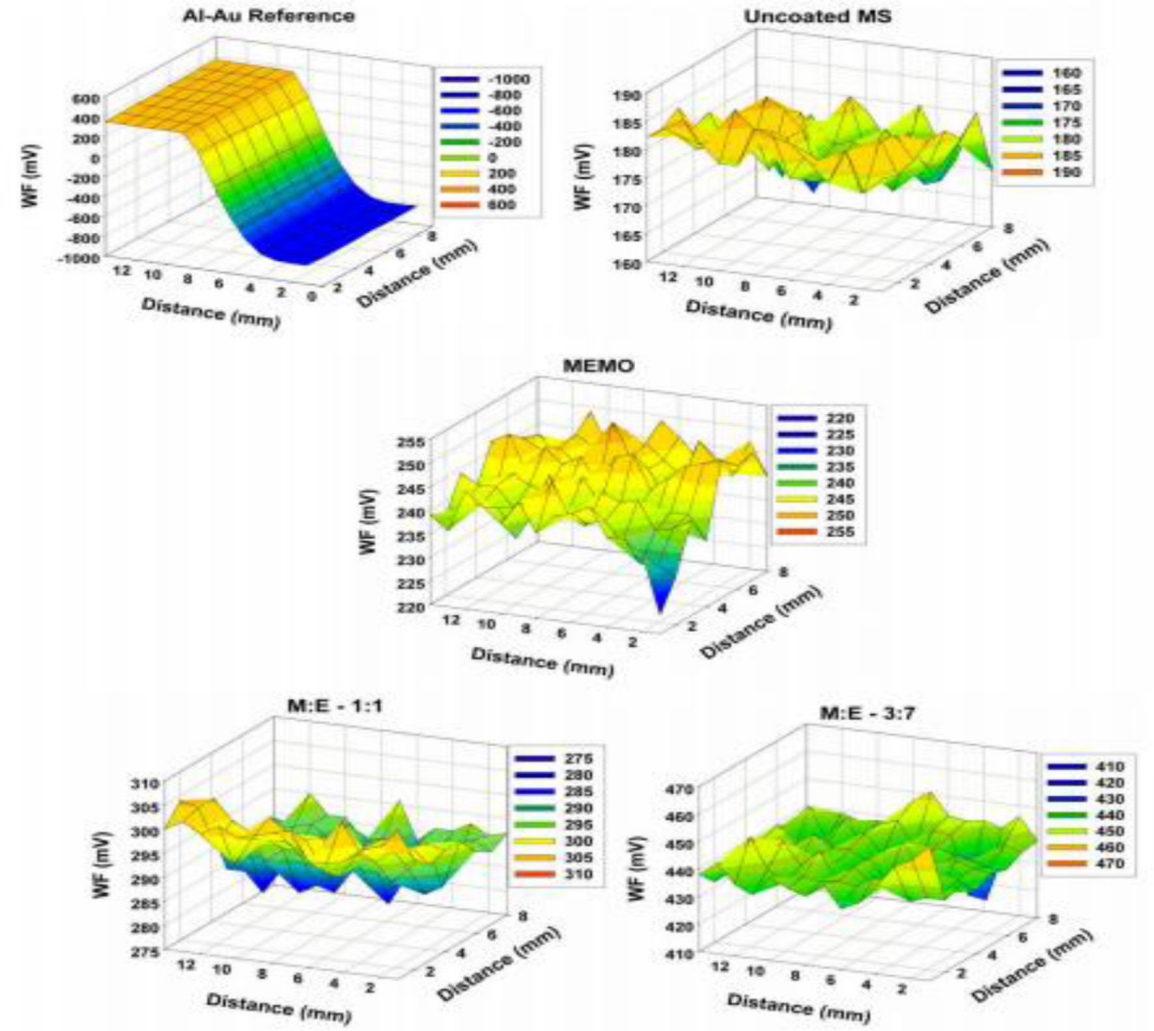
SKP map of pristine: uncoated mild steel (MS), 3-[(methacryloyloxy)propyl] trimethoxysilane (MEMO): 2-(methacryloyloxy) ethyl phosphate (EGMP) M:E 1:1 and M:E 3:7 samples and gold on Al as reference. Reprinted with permission (Kannan et al., 2010). Copyright 2010, Elsevier.

Scanning Kelvin probe (SKP) analysis is one of the advanced characterization methods to evaluate the interfacial strength and corrosion property. SKP is most suitable for non-contact assessments of work function (WF) of the coated or uncoated metal or non-mental interface. The value of work function depends on the type of the materials treated or fabricated. Kannan et al. (2010) fabricated sol-gel derived hybrid coating on mild steel (MS) substrate and established a correlation between WF and corrosion potential through potentiodynamic polarization curves and SKP analysis (Figure 11). Amongst various ratios of methacrylate and phosphated copolymers, M:E 3:7 showed best protection ability and best adhesion strength between the coating and the substrate which leads to improved corrosion resistance due to the strong interaction between substrate and coating. Therefore, SKP could be a promising characterization tool to confirm the results of biocorrosion test methods of Mg based alloys.

**Figure 12:**
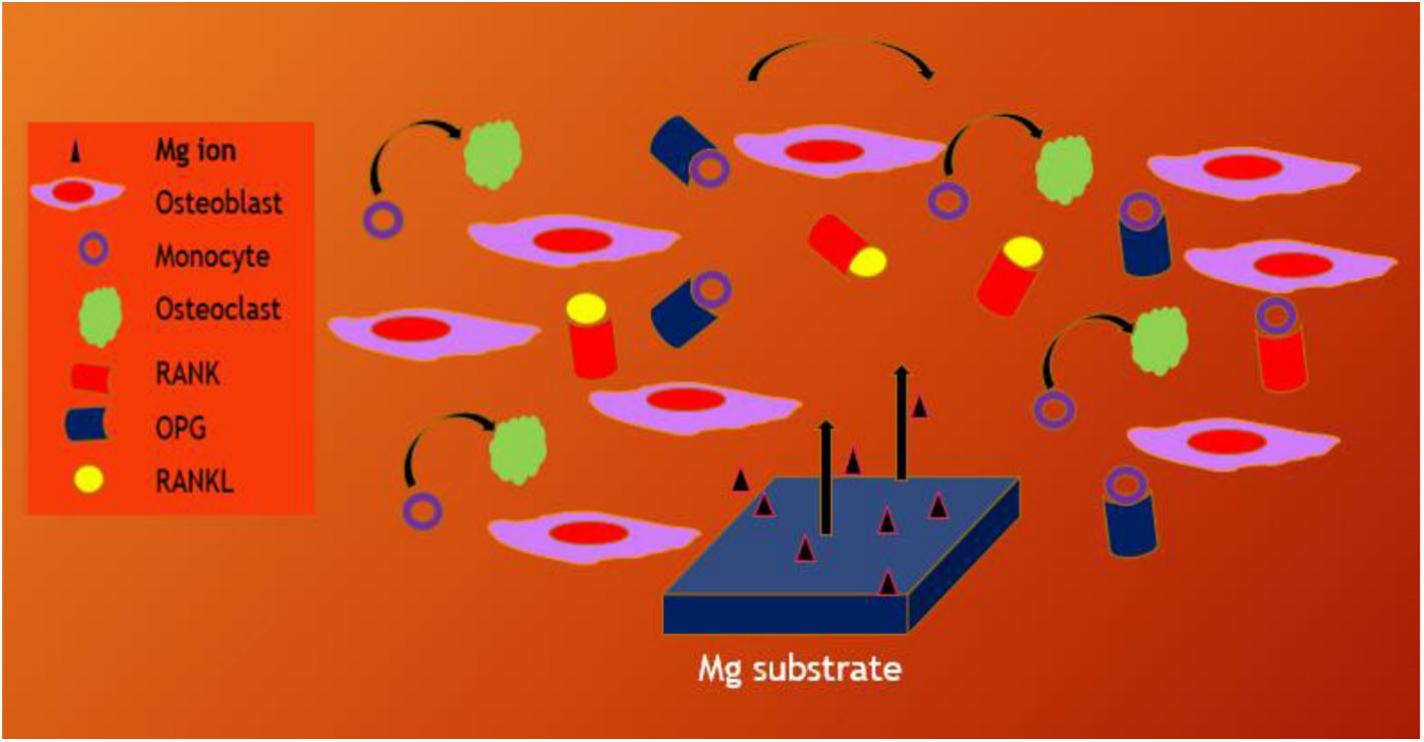
Schematic illustration of bone remodeling on Mg through signaling pathways in the microenvironment regulating the cross talk of osteoblast to osteoclast.

Whenever untreated Mg substrates are exposed to the physiological environment, they rapidly degrade due to chemical reactivity and hence, pretreatment or coating strategies are adopted to control the rate of Mg dissolution. Huge amount of Mg^2+^ dissolution prevents the bone generation or remodeling process. In case of CaP coated Mg alloy substrate, the differentiation of osteoclast from monocyte is prevented due to the dissolution of Mg^2+^ from CaP nanoparticles and reduced osteoclast activity too (Blum et al., 2017; Kim et al., 2017). However, the bone remodeling processes are controlled by signaling pathway such as OPG/RANKL/RANK, estrogen, Wnt signaling pathway, kinase signal transduction pathway and so on (Indran et al., 2016). It is an important approach to medicate the osteoclast-related diseases including peri-prosthetic osteolysis by decreasing bone resorption. Li et al. (2019) fabricated bilayer coating with zoledronic acid (ZA) and CaP on Mg-Sr substrate to promote the osteogenesis properties of the implant material. They reported that the bilayer coated Mg-Sr substrate could control the osteoclastogenesis and osteogenesis NF-κB and estrogen receptor α (ERα) signaling pathway and increasing the ratio of receptor activator of nuclear factor kappa-B ligand (RANKL): OPG, the substrate could control the cross talk of osteoclast-osteoblast in co-culture environment using the RANK/RANKL/OPG signaling pathway that could be stimulating the bone remodeling process as shown in Figure 12. Moreover, osteogenic response of biodegradable Mg–Nd–Zn–Zr substrate was stimulated by the Akt/TLR4/PI3K signaling pathway (Wang et al., 2020).

### Biocompatibility of Coatings

**Figure 13:**
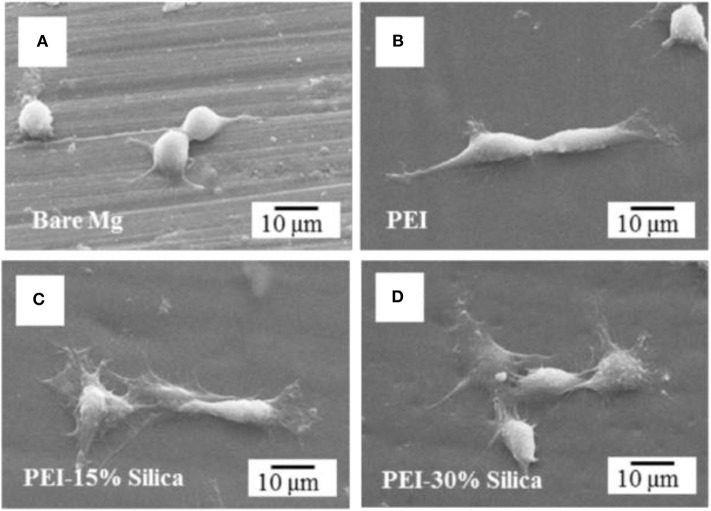
Osteoblastic cell morphology of hybrid coating on Mg sample. **(A)** Bare Mg, **(B)** pure PEI, **(C)** PEI-15% silica, and **(D)** PEI-30% silica coated Mg specimens after 6 h cell attachment. Reprinted with permission (Kang et al., 2016). Copyright 2016, IOP publishing.

The coating combinations such as ceramic/polymer, ceramic/metal, ceramic/ceramic, and even more than two or three constituents could be suitable strategy to mitigate the constraints of using Mg alloys for orthopedic applications. So far, numerous HA-coated composite coatings on the metal implants such as Mg and its alloys, Ti and its alloys have been fabricated due to their outstanding bioactivity and biocompatibility of HA. However, such different method to assess the biocompatibility of implants, cytotoxicity is one of the key indicators for speedily screening the biocompatibility of the biomaterials. The long-term cell response and viability of the uncoated and coated surface of the substrate can be assessed by the osteogenesis and cell adsorption. For example, HA coating can enhance osteointegration and cell viability by dissolving the coating in the biological environment as it has similar composition of natural bone. The effects of the material or coating properties on cell growth, cell attachment, and proliferation thus can be observed through a morphology and the biocompatibility of the treated surface or implant can be assessed. Kang et al. (2016) synthesized hybrid coatings on Mg alloys to investigate biocompatibility and corrosion resistance. The hybrid coated Mg substrate significantly enhances the tissue interfacial ability because of maintaining good adhesion strength and improving hydrophilicity. The authors also suggested that PEI-silica hybrid coated Mg alloy could be promising orthopedic implant by enhancing biocompatibility and corrosion resistance. Cell morphology of hybrid coated Mg and bare Mg has been represented in Figure 13 where pre-osteoblast cell MC3T3-E1 was cultured in alpha minimum essential medium (α-MEM) supplemented with 1% penicillin-streptomycin and 5% fetal bovine serum (FBS) with 5% CO_2_ in a humid incubator at 37°C for 6 h. After 6 h of culture, only a small number of cells with an almost round shape were attached with nominal spreading on the surface of bare Mg sample as seen in Figure 13A. However, in Figures 13B–D, significant cell attachment is noticed on the PEI and PEI-silica coated Mg surfaces. Notably, the cells became less spread out on the pure PEI coating layer whereas on the surface of the PEI-silica hybrid coating layer cells were observed more spread out and flattened.

**Figure 14:**
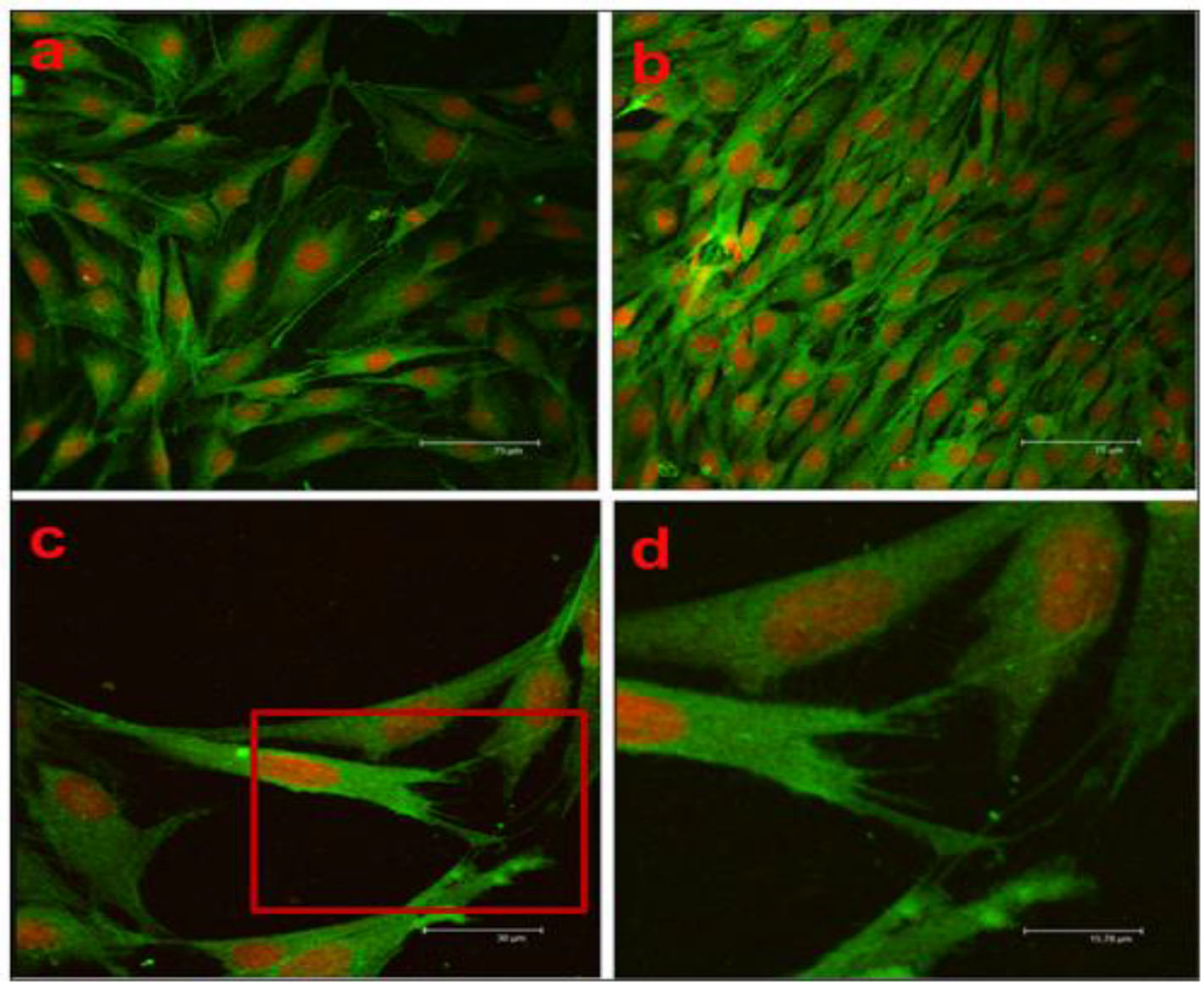
Confocal microscopy of scaffolds: **(a)** PA66 scaffold, **(b)** n-HA/GF/PA66 scaffold (X200), **(c)** n-HA/GF/PA66 scaffold (X500), and **(d)** n-HA/GF/PA66 scaffold (X1000) after 4d cell culture. Reprinted with permission (Su et al., 2013). Copyright 2013, Elsevier.

During the breakdown of the coating, if there is an increase in pH and H_2_ gas, then it leads to enhanced corrosion. Such faster degradation rate makes the substrate unsuitable for cell adhesion and proliferation. A biocompatible coating is expected to promote the cellular behavior. Confocal micrograph is an advanced characterization tool to show the images of cells attachment on the surface of the substrate. A confocal microscopy study was performed on the scaffolds fabricated with nano-hydroxyapatite (nHA)/glass fiber (GF)/polyamide 66 (PA) and MC3T3-E1 cells were seeded to observe the cell attachment, proliferation, and distribution on the implant surface (Su et al., 2013). In Figure 14, after 4d of cell culture, cells (green) indicated a longer spindle-shaped morphology, and the nuclei (red) indicated round or elliptical. The fluorescence staining revealed the cells were well-proliferated and distributed. For this cell culture study, PA66 scaffold considered as negative control and it is found that the cells are tightly attached on the surface of the n-HA/GF/PA66 scaffold. It is also demonstrated that n-HA/GF/PA66 scaffold exhibited better cell adhesion characteristics and better biocompatibility compared to PA66 scaffold due to bioactive coating and nanometer surface effect. Bioinert and ceramic like coatings such as HA and TiO_2_ are beneficial for cell attachment and proliferation. For instance, a composite coating with HA/TiO_2_ was fabricated on Mg substrate to reduce corrosion rate and improve biocompatibility (Amaravathy et al., 2014). It is reported that the composite coated Mg substrate exhibited best corrosion resistance and the cell viability and fluorescence images showed excellent cells attachment and differentiation on the composite coated surface as compared to uncoated Mg sample due to superior surface topography and efficient control of coating degradation. In addition, another cell culture study with MG63 osteoblast like cell performed on AZ31 Mg alloys modified with Ta_2_O_5_ reported that the cells were significantly attached and proliferated on the modified surface due to the synergistic beneficial effect of Ta_2_O_5_ (Madhankumar et al., 2014). The modified surface exhibited adequate corrosion and wear protection with excellent biocompatibility as compared to untreated substrate. The biocompatibility of different coatings is summarized in the Table 3.

**Table 3:** Biocompatibility of different types of coating.

**Substrate**	**Coating type**	**Coating methods**	**Cell line**	**Biocompatibility**	**References**
Mg	HA	Immersion	MCT3T3-E1	+	Kim et al., 2014
AZ31	PDA/HA	Immersion	L-929	+	Lin et al., 2015
AZ91	CaP/PLLA	EDP	–	+	Kannan and Liyanaarachchi, 2013
Mg-1Li-1Ca	MAO/PLLA	MAO+ Dipping	MCT3T3-E1	++	Zeng et al., 2016
AZ31/Mg_4_Y	PLGA	Dipping	MCT3T3-E1		Ostrowski et al., 2013
AZ31	PCL	Electrospinning	L6	+	Hanas et al., 2016
AZ31B	FHA/MAO	Hydrothermal	MCT3T3-E1	+	Yu et al., 2019
AZ31/ZK41	Hybrid	Sol-gel	Fibroblast	++	Córdoba et al., 2019

*+, good; ++, excellent*.

**Figure 15:**
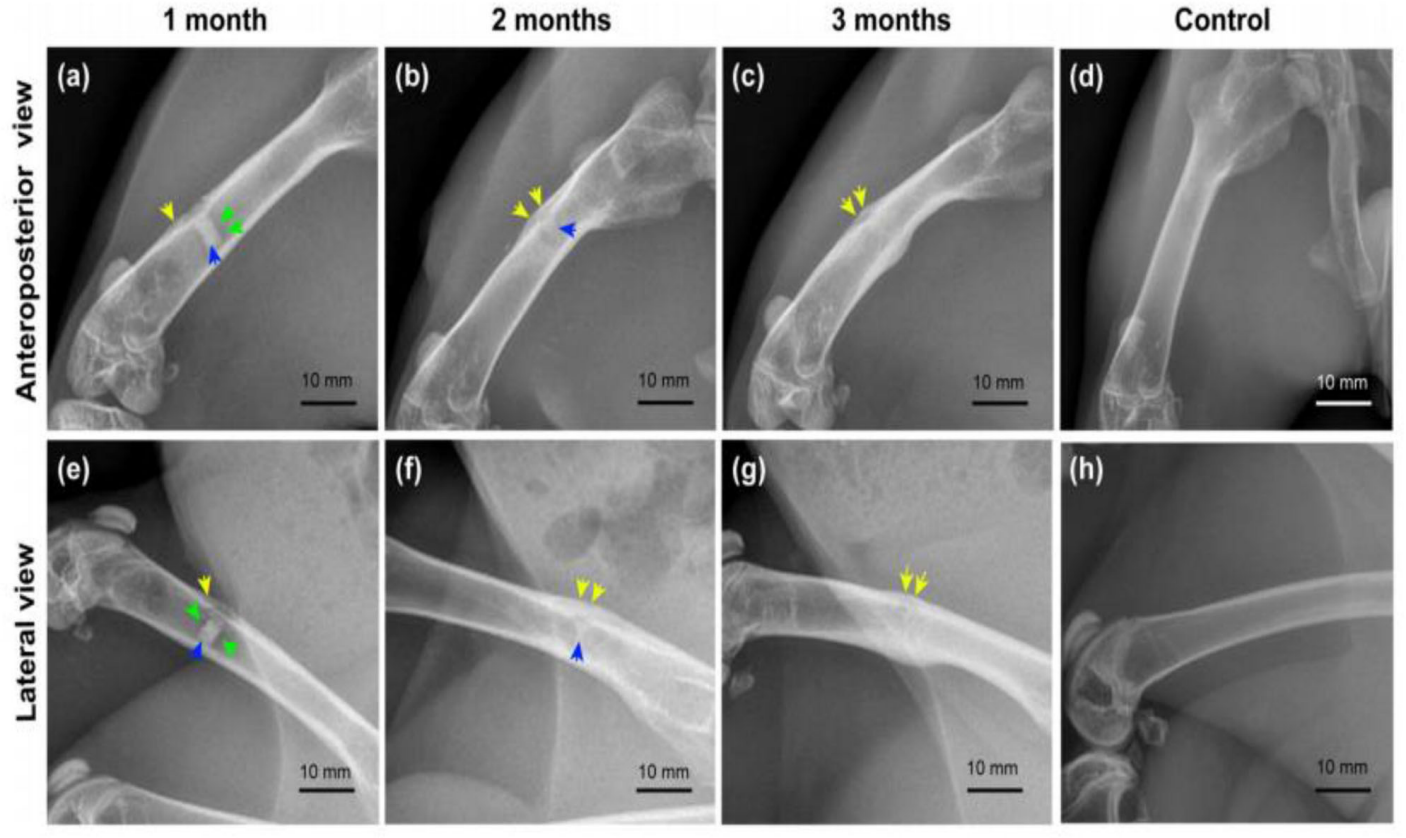
Radiography examination of composite coated Mg alloy implant in the femur of rabbit for different time periods. Yellow arrows, periosteal reaction/callus; green arrows, hydrogen bubble; blue arrows, residual implant. **(a–c)** Anteroposterior view of femurs in the experimental group; **(e–g)** lateral view of femurs in the experimental group; **(d,h)** anteroposterior and lateral view of femurs in the control group (Liu et al., 2020).

Both *in vitro* and *in vivo* assessments are very important to evaluate the performance of the implant materials for biomedical applications. Interfacial engineering by fabricating coating on the Mg alloys such as CaP or other biocompatible coating could improve the *in vitro* and *in vivo* performance. However, CaP coatings have dual benefit, for example, it can delay the corrosion making a layer on substrate surface, thus, enhance corrosion resistance, on the other hand, Ca and P are bone mineral composition thereby improve biocompatibility of implant materials. *In vitro* assessment confirmed that the Sr doped CaP coating ZK60 Mg alloy substrate enhanced corrosion resistance, improved bioactivity, promoted cell adhesion and proliferation and the *in vivo* study revealed that Mg alloy improved biocompatibility and excellent osteointegration through higher bone regeneration post-implantation in a rabbit model (Makkar et al., 2020). Moreover, another *in vitro* study was performed on composite (gelatin/dopamine /rhBMP-2– coated β-TCP/Mg-Zn) coated Mg substrate to enhance biocorrosion and osteoconductivity (Liu et al., 2020). *In vitro* study revealed that the composite coated substrate not only improved biodegradation but also cell proliferation. The radiography examination revealed that the corrosion begun and observed some defects at the early stage of implantation, but the defects were repaired with increasing implantation time as shown in Figure 15. In Figures 15b,f, the implant shadow was reduced after 2 months as compared to the Figures 15a,e after 1 month of implantation. After 3 months of implantation, there is no implant shadow and it completely disappeared in Figures 15c,g. Therefore, the *in vivo* results suggest that the composite coated Mg alloy substrate significantly promoted bone regeneration and reduced biodegradation.

## Conclusions and Future Outlook

Although the great advantages of Mg-based alloys such as biocompatibility and biodegradability, but their fast corrosion and rapid degradation leading to the formation of H_2_ gas are the great constraints for using them in biomedical applications. Surface and interface modifications with polymers and hybrids are the promising approaches that can effectively modify the surface properties of the Mg implants. There are different strategies including surface modification using chemical treatment or coatings by single layer or multilayers, addition of new alloying elements during alloy fabrication, composite materials have been adopted to control the corrosion and degradation in the physiological environment. The major disadvantages of many coatings are poor adhesion strength. Often the insufficient adhesion strength leads to the coating delamination by mechanical shear, which is a significant concern in clinical application. Therefore, new type of surface treatment like hybrid coating combining the multiple layers of anodization, HA, biomimetic coating with self-assembling protein, silk fibroin etc. could be promising choice to overcome some of the above constraints. So, firstly, hybrid coating can easily produce a denser and uniform coating layer without any microcracks that offer strong protection against corrosion in the biological environment. Secondly, the hybrid coating has flexibility to include inhibitors and pigments as anti-corrosion additives, thus, improve corrosion protection ability, and cell adhesion and proliferation. The interface properties of such hybrid coatings such as adhesion, delamination etc. for Mg metal are the important factors for the quality and stability of the coatings. Only a few reports detail adhesion test for evaluating the stability and feasibility of polymer or hybrid coatings. Other important parameters like pH, solvents/electrolytes, or hydrolysis of coating or Mg, defect formation, etc. are all crucial parameters for the comprehensive understanding of the coating/film formation on Mg alloys and play an important role in large scale production of Mg based implants. Also new hybrid materials with multiple phase systems hold promise for Mg implant coating with enhanced mechanical properties, low hydrolysis rate, and reduced toxicity. In addition, the combination of hydrophobic/hydrophilic components with desired mechanical characteristics or their layer-by-layer assembly can also be incorporated in future for coating systems, to achieve stable, anti-corrosive, and biocompatible coatings for orthopedic applications. Also, the HA coating on the Mg alloys exhibits the good biocompatibility, good corrosion resistance, although sometimes, it produces an uneven coating thickness and non-uniform morphology, making them susceptible to corrosion. Therefore, hybrid coatings with multilayers including chemical treatment, HA coating, and protein-based polymer coatings can be a promising approach to address these limitations of Mg-based alloys for promoting their mechanical integrity, biocompatibility, bioactivity, and corrosion performance for biomedical applications.

## Author Contributions

MR, NR, and ND did conceptualization, design of the review, and reviewed and edited the article. MR prepared the initial draft of the manuscript under the supervision of NR. NR wrote part of the article.

## Conflict of Interest

The authors declare that the research was conducted in the absence of any commercial or financial relationships that could be construed as a potential conflict of interest.

## Abbreviations

α-MEM: alpha minimum essential medium
BCP: Bi-phasic calcium phosphate
CaP: Calcium phosphate
Co-Cr: cobalt-chromium
CA: contact angle
DCPD: dicalcium phosphate dihydrate
EIS: electrochemical impedance spectroscopy
ECM: extracellular matrix
ERK: extracellular signal-regulated kinase
FBS: fetal bovine serum
FHA: fluorinated HA
FDA: Food and Drug Administration
GO: graphene oxide
GF: glass fiber
HA: hydroxyapatite
H_2_: hydrogen
HPM: high purity magnesium
LBL: layer by layer
MS: mild steel
Mg: magnesium
nHA: nano-hydroxyapatite
OI: osseointegration
PEO: plasma electrolytic oxidation
PA: polyamide
PGA: polyglycolic acid
PLA: polylactic acid
PAA: polyacrylic acid
PCL: polycaprolactone
PHB: polyhdroxubutyrate
PEI: polyether imide
Sr: strontium
SF: silk fibroin
SKP: Scanning Kelvin probe
Ti: titanium
TCP: tricalcium phosphate
TA: tannic acid
WF: work function
MEMO, 3-[methacryloyloxy: propyl] trimethoxysilane.
